# What Are Priorities for Deprescribing for Elderly Patients? Capturing the Voice of Practitioners: A Modified Delphi Process

**DOI:** 10.1371/journal.pone.0122246

**Published:** 2015-04-07

**Authors:** Barbara Farrell, Corey Tsang, Lalitha Raman-Wilms, Hannah Irving, James Conklin, Kevin Pottie

**Affiliations:** 1 Bruyère Research Institute, Ottawa, Canada; 2 Department of Family Medicine, University of Ottawa, Ottawa, Canada; 3 School of Pharmacy, University of Waterloo, Waterloo, Canada; 4 Leslie Dan Faculty of Pharmacy, University of Toronto, Toronto, Canada; 5 Department of Applied Human Sciences, Concordia University, Montreal, Canada; Örebro University, SWEDEN

## Abstract

Polypharmacy and inappropriate medication use among older adults contribute to adverse drug reactions, falls, cognitive impairment, noncompliance, hospitalization and mortality. While deprescribing - tapering, reducing or stopping a medication - is feasible and relatively safe, clinicians find it difficult to carry out. Deprescribing guidelines would facilitate this process. The aim of this paper is to identify and prioritize medication classes where evidence-based deprescribing guidelines would be of benefit to clinicians. A modified Delphi approach included a literature review to identify potentially inappropriate medications for the elderly, an expert panel to develop survey content and three survey rounds to seek consensus on priorities. Panel participants included three pharmacists, two family physicians and one social scientist. Sixty-five Canadian geriatrics experts (36 pharmacists, 19 physicians and 10 nurse practitioners) participated in the survey. Twenty-nine drugs/drug classes were included in the first survey with 14 reaching the required (≥ 70%) level of consensus, and 2 new drug classes added from qualitative comments. Fifty-three participants completed round two, and 47 participants completed round three. The final five priorities were benzodiazepines, atypical antipsychotics, statins, tricyclic antidepressants, and proton pump inhibitors; nine other drug classes were also identified as being in need of evidence-based deprescribing guidelines. The Delphi consensus process identified five priority drug classes for which expert clinicians felt guidance is needed for deprescribing. The classes of drugs that emerged strongly from the rankings dealt with mental health, cardiovascular, gastroenterological, and neurological conditions. The results suggest that deprescribing and overtreatment occurs through the full spectrum of primary care, and that evidence-based deprescribing guidelines are a priority in the care of the elderly.

## Introduction

Overdiagnosis and overtreatment are potentially harmful and expensive [1–3]. In 2012, BMJ launched a series of articles exploring the potential for overdiagnosis in specific conditions. The call for new research in this field led to the inaugural Preventing Overdiagnosis conference in 2013 [4]. Linked to overdiagnosis is the challenge of overtreatment, and in particular polypharmacy in the elderly. This paper provides direction to those seeking to develop approaches to reducing overtreatment in the elderly.

Polypharmacy and inappropriate medication use among older adults are known to contribute to adverse drug reactions, falls, cognitive impairment, noncompliance, hospitalization and mortality [5–11]. While deprescribing—the act of tapering, reducing or stopping a medication—has been shown in small studies to be feasible and relatively safe [12–14], clinicians continue to find it difficult to stop medications [15,16]. Barriers include difficulty making decisions to stop medications (both from the clinician and patient perspective), worry about stopping medications started by others, limited knowledge about how to stop medications, and concern about medication withdrawal effects [15]. In addition, clinicians feel pressured to prescribe according to clinical guidelines but recognize that such guidelines are rarely based on evidence from studies in older populations and rarely address modifying clinical targets with advancing age or care goals [15,17,18].

Innovative approaches are needed to address these barriers in order to limit the negative impact of polypharmacy on our older population. Such approaches should facilitate decision-making about stopping a medication and provide clear recommendations for tapering and monitoring impact to ensure safety and effectiveness of the process. To achieve this, the Ontario (Canada) Ministry of Health and Long-Term Care has supported the systematic development and testing of a series of evidence-based guidelines for deprescribing.

Given the large number of drug classes felt to be potentially inappropriate or risky in the elderly [19,20], determining priorities for developing such guidelines is challenging. In keeping with initiating a successful guideline enterprise and seeking input from relevant professional groups, we elected to conduct a priority setting process to identify, balance and rank priorities by expert stakeholders [21,22]. The aim of this Delphi consensus process was to engage physicians, pharmacists and nurses in identifying and prioritizing medication classes where evidence-based deprescribing guidelines would be of benefit to clinicians.

## Participants and Methods

### Study design

A modified Delphi approach [23], beginning with a literature review to identify potentially inappropriate medications for the elderly and existing approaches to deprescribing, followed by expert panel discussion and three rounds of surveys, was used to generate and achieve consensus among experts regarding priorities for deprescribing guidelines for the elderly.

### Ethics approval

Ethics approval was obtained from the following Research Ethics Boards: Bruyère Continuing Care and Ottawa Health Science Network (Ottawa, Ontario), Concordia University (Montreal, Quebec), University of Toronto (Toronto, Ontario) and University of Waterloo (Waterloo, Ontario). All participants provided informed consent with each survey iteration.

### Delphi working group

Six members of the research team, which included two family physicians and three pharmacists, all with expertise in geriatrics, and a social scientist with expertise in evaluating change, met in person in July 2013. The group reviewed literature and reports outlining the prevalence and impact of polypharmacy and potentially inappropriate medication use (e.g. propensity for adverse events and related hospital admissions, and cost-related impacts) [5,24–33], as well as current approaches to deprescribing [12–14,31,34–41]. They next developed a list of drugs and drug classes for experts to consider in recommending priorities for deprescribing guidelines (S1 File).

### Delphi expert panel (survey participants)

Canadian clinical experts from medicine, pharmacy and nursing were purposely identified using the following inclusion criteria: a) geriatrics expertise and/or b) academic appointment in teaching and/or c) research in the area of geriatric pharmacotherapy. Research team members considered those who had a background and experience with polypharmacy management and deprescribing in the elderly, ensuring that participants were highly trained and knowledgeable about the target subject [42]. We used a pragmatic approach to select primary care experts who were in touch with their clinician communities and in tune with emerging changes in their professions for the Delphi consensus. We included professionals in our personal networks, those who had contacted us with interest in the project as well as faculty members listed in applicable departments at universities across Canada. E-mail invitations were sent to each expert to determine interest and to explain the time commitment involved in participating in the Delphi process.

### Survey administration and analysis

Three rounds of surveys were administered via Fluid Surveys (http://fluidsurveys.com/) from November 2013 to February 2014. Each survey was live for two weeks; two reminders were sent. The definition of consensus was determined before the analysis of each round, by the investigator team, and in consultation with a statistician. Investigators were blinded to the results during analysis. Specific definitions of consensus are explained in each round described below.

### Round one

Participants’ demographics such as health professional background, type of practice, age range and gender were collected. Participants were provided with a clinical scenario (S1 File) and the four criteria commonly used in guideline development (adapted from the GRADE guideline development approach) [43]: 1) benefits vs. harms of medication therapy; 2) certainty of estimate of effects; 3) patient preference and values; and 4) feasibility and cost. They were also asked to consider the need for guidance in relation to both stopping the medication and managing the impact of stopping the medication. They rated each drug/drug class on a 5-point Likert-type scale indicating the ‘usefulness’ of an evidence-based ‘deprescribing guideline’: “1—Definitely not useful”, “2—Likely not useful”, “3—Might be useful”, “4—Probably useful”, “5—Definitely useful”. Free-text boxes were included for each drug/drug class to capture optional comments; these were reviewed by the research team to determine whether such comments could assist in understanding similarities or differences in rating of the drugs/drug classes. Microsoft Excel was used to run descriptive statistical analysis, including mean and standard deviation. A priori, investigators determined that drugs or drug classes identified by ≥70% of participants as either probably or definitely useful would be retained for inclusion in round two [44]. Participants were also asked to provide the names of drugs or drug classes felt to be a priority but that were missing from the expert panel generated list. If more than 10% of respondents included a new drug or drug class, it was added to the list for round two. See S1 File for a copy of the round one survey.

### Round two

**Fig 1:**
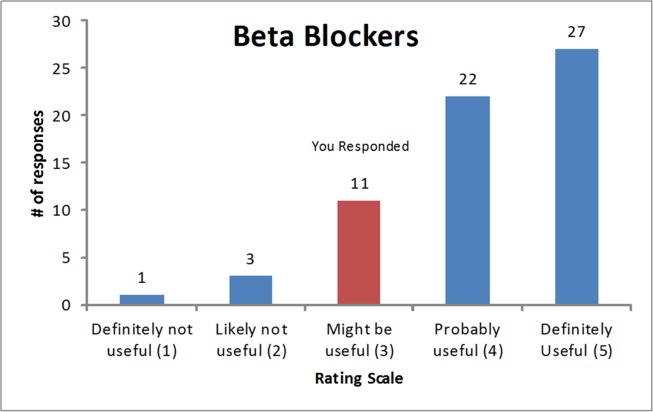
Example histogram showing overall first round and personal results.

Round two of the survey consisted of two sections. In the first section, participants were asked to use the same instructions as for round one to rate two new drug classes added as a result of the round one survey; investigators agreed these new drug classes would be included in round three if ≥70% of participants rated them as either probably or definitely useful. In the second section, participants received an individualized e-mail containing histograms for each drug/drug class showing overall round one results [44] and indicating their personal rating (Fig 1). For new drugs added from the results of round one, no histograms were available. Experts were asked to rank each drug/drug class in order (from 1—highest priority to 16—lowest priority) with respect to the need for a deprescribing guideline; this approach was chosen in order to induce participants to make choices about priorities [45]. A mean rank (and standard deviation) was calculated for each drug/drug class [45]. Kendall’s W coefficient of concordance was calculated for the overall group of respondents and within each health care professional group to assist in determining whether consensus (W = 0.7) had been reached and whether a third round was warranted [46,47]. For their top 5 choices, participants were required to complete a free-text section providing justification for ranking these drugs/drug classes as highest priority. This qualitative text was analyzed by a research team member using simple content analysis[48,49] to identify themes that arose as participants considered ranking priorities; Each research team member then read the qualitative comments and themes independently and discussed them together with the initial coder at a team meeting, using group discussion to verify the themes and resolve disagreements. See S2 File for a copy of the round two survey.

### Round three

Round three of the survey included drug classes only. Two individual drugs were removed from the list in an effort to establish priorities for more widely applicable guidelines. All results from round two were presented to participants in the order of their mean rank. Participants were asked to consider the following criteria generated from respondent comments in round two: 1) uncertainty of benefit in the elderly; 2) high risk of harms in the elderly; 3) availability of suitable alternatives; 4) potentially high impact of a deprescribing guideline for the elderly; and 5) feasibility for guideline development (i.e. an adequate amount of literature to create an evidence-based guideline). Using these criteria, they were asked to identify only the top five drug classes that they felt had an urgent and clear need for a deprescribing guideline. Similar to round two, mean rank with standard deviation was calculated. In addition, the number of respondents who chose each drug class as one of their top five choices was calculated [50]. Investigators planned to consider both results: mean rank and number of respondents choosing each drug class, in determining consensus about priority drug classes. No free-text option was provided and no qualitative data was gathered during this round. See S3 File for a copy of the round three survey.

## Results

**Fig 2:**
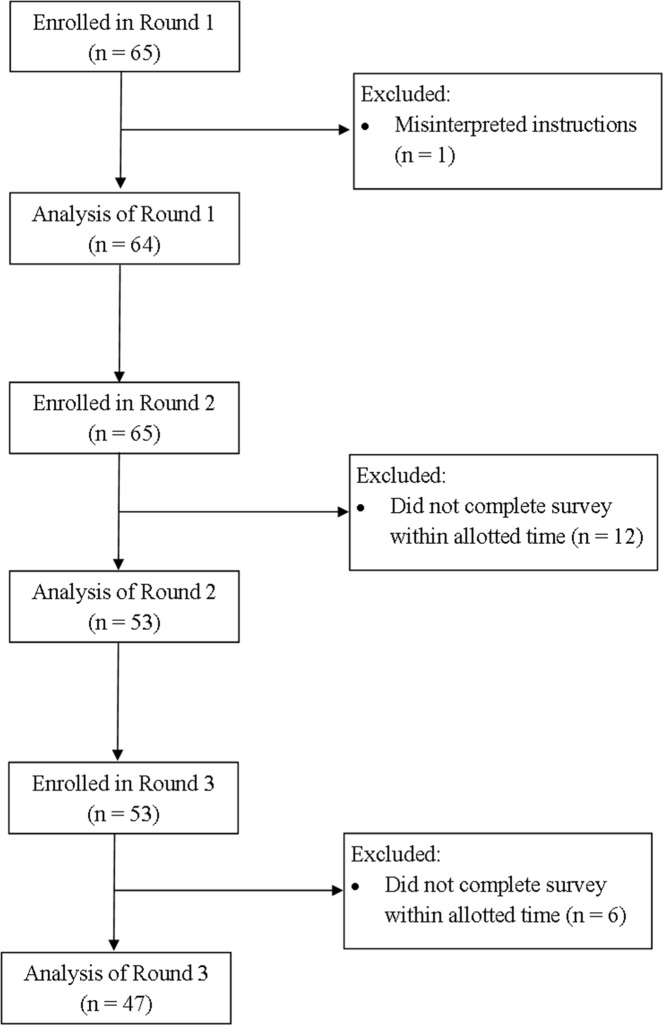
Participant flow diagram through three rounds of the Delphi consensus process.

The expert panel identified 29 drug/drug classes for inclusion in the first survey. Sixty-five experts agreed to participate in the Delphi process, including 8 geriatricians, 11 family physicians, 36 pharmacists and 10 nurse practitioners representing eight of the 10 Canadian provinces. (see Table 1 for survey participant characteristics and Fig 2 for participant flow through the Delphi process)

**Table 1:** Characteristics of Survey Participants.

	Round 1 (n = 64)^a^	Round 2 (n = 53)	Round 3 (n = 47)
**Profession**			
Pharmacist	35 (55%)	34 (64%)	32 (68%)
Family Physician	11 (17%)	7 (13%)	5 (11%)
Geriatrician	8 (12%)	5 (9%)	4 (9%)
Nurse Practitioner	10 (16%)	7 (13%)	6 (13%)
**Years of Practice**			
Less than 5	5 (8%)	5 (9%)	4 (9%)
5–9	9 (14%)	4 (8%)	3 (6%)
10–14	18 (28%)	17 (32%)	16 (34%)
15–19	10 (16%)	7 (13%)	7 (15%)
20–24	10 (16%)	8 (15%)	7 (15%)
25+	12 (19%)	12 (23%)	10 (21%)
**Practice Type**			
Long-term care	8 (12%)	7 (13%)	5 (11%)
Primary health care	23 (36%)	20 (38%)	19 (40%)
Other (primarily hospital and specialty clinics)	33 (52%)	26 (49%)	23 (49%)
**Gender**			
Male	16 (25%)	15 (28%)	14 (30%)
Female	48 (75%)	38 (72%)	33 (70%)
**Age Range**			
34 and under	7 (11%)	7 (13%)	5 (11%)
35–44	20 (31%)	18 (34%)	17 (36%)
45–54	23 (36%)	17 (32%)	16 (34%)
55–64	13 (20%)	11 (21%)	9 (19%)
65+	1 (2%)	0 (0%)	0 (0%)
**Province**			
BC	1 (2%)	1 (2%)	1 (2%)
AB	6 (9%)	6 (11%)	6 (13%)
SK	2 (3%)	2 (4%)	2 (4%)
MB	1 (2%)	1 (2%)	1 (2%)
ON	48 (75%)	36 (68%)	31 (66%)
QC	3 (4%)	3 (6%)	3 (6%)
NB	1 (2%)	2 (4%)	2 (4%)
NS	2 (3%)	2 (4%)	1 (2%)

^a^one pharmacist response deleted due to incorrect use of rating scale

### Round one

Sixty-four participants correctly completed the first survey; one participant’s responses were removed from the analysis after they contacted us to indicate they had applied the rating scale in the opposite order for some responses. Fourteen of the 29 drug/drug classes reached ≥ 70% level of consensus as being probably or definitely useful and are listed in Table 2 in order of the level of consensus achieved (in ties, the drug or drug class with the higher mean rating is ranked higher). Two new drug classes (anticonvulsants and bisphosphonates) were identified as priorities by >10% of participants in round one. The top five rated drugs/drug classes included benzodiazepines followed by atypical antipsychotics, proton pump inhibitors, typical antipsychotics and zopiclone.

**Table 2:** Round One Ranking: Drug/drug classes identified by ≥ 70% of participants as probably or definitely useful.

**Drug/Drug class**	**Number and percent of participants identifying that a deprescribing guideline would be probably or definitely useful**	**Mean rating**	**Standard Deviation**
**1. Benzodiazepines**	59/64 (92%)	4.63	0.96
**2. Atypical antipsychotics**	59/64 (92%)	4.55	0.77
**3. Proton-pump inhibitors**	56/64 (88%)	4.44	0.75
**4. Typical antipsychotics**	56/64 (88%)	4.38	0.86
**5. Zopiclone**	55/64 (86%)	4.41	0.86
**6. Opioids**	53/64 (83%)	4.22	0.80
**7. Statins**	52/64 (81%)	4.25	0.94
**8. Urinary anticholinergics**	52/64 (81%)	4.19	0.88
**9. Tricyclic antidepressants**	49/64 (77%)	4.17	0.94
**10. Beta blockers**	49/64 (77%)	4.11	0.95
**11. Cholinesterase inhibitors**	47/64 (73%)	4.16	0.88
**12. Antiplatelets**	47/64 (73%)	3.94	1.04
**13. Selective serotonin reuptake inhibitors**	46/64 (72%)	3.98	0.93
**14. Trazodone**	46/64 (72%)	4.09	0.84

Respondents’ optional comments indicated strong support for a number of classes as priorities for deprescribing guidelines, as well as divergence of opinion for a number of classes. Strong support is exemplified through comments made in support of rating proton pump inhibitors as higher priority: “SOOOOO [*sic*] over used. When and how to stop would be useful for folks!” and “Please please please provide help on why we don’t need to use these. MDs are reluctant to taper or DC [discontinue] as there is history of PUD [peptic ulcer disease] 20 years ago…” Divergence in opinion is exemplified with this comment from one who rated selective serotonin reuptake inhibitors as low priority: “these drugs are underused, and in general, it is likely that patients need ongoing therapy as first episode of depression in older age is less common than adult onset depression”, and this comment from a second respondent who rated the class as high priority indicating they are “always questioning value of antidepressants in patients over 85 years of age.” Similarly, with regard to prioritizing cholinesterase inhibitors for a guideline, one respondent stated “Please! With patient/family handouts too. Unfortunately, [journal name] etc. have been publishing papers that support using these, but previous cost analyses showed otherwise,” whereas another respondent stated “These drugs are underused and though there is uncertainty regarding when to stop, they are lower priority compared to others.”

From the general comments section, these quotes struck the research team as important to consider: “Deprescribing should like be a component of all treatment guidelines. To date it has not been given the attention it deserves. I feel focusing in on a few drugs, developing the methods for deprescribing guidelines and getting a few guidelines into play is what is needed to catalyze a larger deprescribing movement.” and “I really think all prevention-oriented meds deserve a deprescribing guide.”

### Round two

The 14 drugs/drug classes and two new drug classes from round one were ranked in round two. Fifty-three of the 65 (82%) round one participants (including the respondent who incorrectly completed round 1 but completed rounds 2 and 3 correctly) completed round two. Twelve participants did not complete the round two ranking within the two week allotted timeframe; of these, we received 2 automatic replies indicating the recipient was away on vacation (over the annual December holiday season). The 16 drugs/drug classes are shown in order of their mean rank in Table 3. This table also includes the subgroup findings within each profession, as well as Kendall’s W coefficient of concordance for each group. Kendall’s W values were low across all respondents, as well as within each health care professional group, demonstrating low agreement among participants on the rank order of drugs/drug classes in round two, and the need for a third survey round.

**Table 3:** Round Two Ranking: Overall and by healthcare profession.

**Overall Rank**	**All (*n* = 53) (Mean Rank; SD) Kendall’s W: 0.179, *p* < 0.001**	**Family Physicians (*n* = 7) (Mean Rank; SD) Kendall’s W: 0.347, *p* = 0.002**	**Geriatricians (*n* = 5) (Mean Rank; SD) Kendall’s W: 0.290, *p* = 0.115**	**Pharmacists (*n* = 34) (Mean Rank; SD) Kendall’s W: 0.215, *p* < 0.001**	**Nurse Practitioners (*n* = 7) (Mean Rank; SD) Kendall’s W: 0.204, *p* = 0.123**
**#1**	Benzodiazepines(3.08;2.84)	Benzodiazepines (3.14;1.88)	Benzodiazepines (3;4)	Benzodiazepines (2.76;2.29)	Tricyclic antidepressants (4.43;2.5)
**#2**	Atypical antipsychotics (5.58;4.15)	Statins (3.86;1.73)	Tricyclic antidepressants (5.6;2.73)	Atypical antipsychotics (4.94;3.75)	Benzodiazepines (4.57;4.24)
**#3**	Tricyclic antidepressants (7.38;3.55)	Proton-pump inhibitors (4.71;3.81)	Urinary anticholinergics (6.2;5)	Typical antipsychotics (6.94;4.58)	Atypical antipsychotics (6;5.04)
**#4**	Typical antipsychotics (7.72;4.6)	Bisphosphonates (6.57;2.72)	Zopiclone (6.8;2.93)	Tricyclic antidepressants (7.53;3.18)	Statins (7;5.35)
**#5**	Statins (7.98;4.49)	Atypical antipsychotics (7.43;3.66)	Atypical antipsychotics (6.8;4.79)	Opioids (8.06;4.63)	Typical antipsychotics (7.29;5.23)
**#6**	Proton-pump Inhibitors (8.04;4.7)	Opioids (8.14;5.89)	Anticonvulsants (7.6;3.38)	Cholinesterase inhibitors (8.32;4.46)	Proton-pump inhibitors (7.86;3.52)
**#7**	Zopiclone (8.51;4.27)	Zopiclone (8.29;3.45)	Typical antipsychotics (8;3.03)	Zopiclone (8.62;4.61)	Selective serotonin reuptake inhibitors (8;4.38)
**#8**	Cholinesterase Inhibitors (8.58;4.54)	Beta blockers (8.57;3.96)	Statins (8.4;2.5)	Proton-pump inhibitors (8.71;4.93)	Cholinesterase inhibitors (9.14;3.8)
**#9**	Opioids (8.62;5.09)	Cholinesterase inhibitors (9.29;4.86)	Proton-pump inhibitors (8.4;3.61)	Statins (8.97;4.39)	Zopiclone (9.43;3.66)
**#10**	Urinary anticholinergics (8.91;4.48)	Urinary anticholinergics (9.43;5.07)	Cholinesterase inhibitors (8.6;5.28)	Urinary anticholinergics (9.06;4.29)	Urinary anticholinergics (9.57;3.62)
**#11**	Selective serotonin reuptake inhibitors (9.53;3.91)	Antiplatelets (9.71;3.45)	Trazodone (9.8;6.05)	Selective serotonin reuptake inhibitors (9.24;3.5)	Beta blockers (9.71;4.37)
**#12**	Bisphosphonates (9.83;3.69)	Trazodone (10;2.73)	Bisphosphonates (10.2;3.06)	Beta blockers (10.06;4.14)	Antiplatelets (10;2)
**#13**	Beta blockers (10;4.07)	Tricyclic antidepressants (10.86;3.36)	Selective serotonin reuptake inhibitors (10.8;3.76)	Anticonvulsants (10.21;4.21)	Opioids (10.29;5.55)
**#14**	Anticonvulsants (10.38;4.32)	Selective serotonin reuptake inhibitors (11.57;4.34)	Opioids (10.8;5.04)	Bisphosphonates (10.26;3.88)	Bisphosphonates (10.71;1.91)
**#15**	Antiplatelets (10.87;3.85)	Typical antipsychotics (11.71;2.31)	Beta blockers (12;1.79)	Antiplatelets (10.97;4.23)	Anticonvulsants (10.86;4.64)
**#16**	Trazodone (11;3.77)	Anticonvulsants (12.71;3.73)	Antiplatelets (13;2.28)	Trazodone (11.35;3.45)	Trazodone (11.14;3.68)

Content analysis of qualitative comments suggested that respondents used five main criteria in making their selections for determining guideline priorities: a) risk of continuing the drug; 2) questions about ongoing indication or benefit of the drug; 3) prevalence of overuse of the drug; 4) challenge in stopping the drug; and 5) the availability of other treatment options. See Table 4 for examples of comments illustrating each criterion.

**Table 4:** Themes identified from content analysis of Round Two comments.

**Theme**	**Representative comment**
Risk of continuing the drug	With respect to: anticonvulsants “There are no guidelines on how to stop this in older adults who had a history of seizure disorder in their youth and now falling in their senior years and worsening cognitively with no seizure activity in decades—it is something very few people feel comfortable stopping yet contributes to worsening of geriatric syndromes.” With respect to: tricyclic antidepressants “find it very problematic in my practice that they are used off label for sleep, gp’s reluctant to ‘mess with their sleep’ despite falls, confusion etc, not wanting to affect their ‘mood’” With respect to: beta-blockers “Elderly are at high risk for accumulation due to changes in PCK; doses rarely get lowered as people age: common to see bradycardia, fatigue and OH—all potentially leading to falls; therefore risk starts to outweigh benefit—especially for frail elderly—therefore, should be a high priority.”
Question about ongoing indication or benefit of the drug	With respect to: selective serotonin reuptake inhibitors “Often prescribed for ‘grief’ or ‘sadness’ related to hospitalization or deconditioning and continued for many years.” With respect to: statins “Many questions about effectiveness given lack of elderly people in trials”
Prevalence of overuse of the drug	With respect to: atypical antipsychotics “affects 30% of residents in long-term care” With respect to: benzodiazepines “despite suggestions in the literature about how to wean people off, clinicians continue to keep people on them.” and “high volume contributor to delirium, falls, hospitalization.” With respect to: proton pump inhibitors “this is such a commonly used medication that is most often stopped with no adverse effect whatsoever!”
Challenge in stopping the drug	With respect to: cholinesterase inhibitors “Many physicians are unaware of the need to taper and realistically, which symptoms should be monitored.” With respect to: statins “We need more info about when these meds are no longer beneficial to a patient. When can we stop statins? It’s easy to stop them (no withdrawal) but when is appropriate?”
Availability of other treatment options	With respect to: opioids “There is harm associated with these medications and their use often results in a prescribing cascade. Often treatment of pain isn’t explored fully with other, safer options before the reaction to start these meds occurs.” With respect to: selective serotonin reuptake inhibitors “Other non-drug approaches might be better for helping people cope with aging.”

In round two, benzodiazepines remained the top priority and atypical antipsychotics remained the second-highest priority for most participants, followed by tricyclic antidepressants, typical antipsychotics and statins. The qualitative comments suggest that those who ranked benzodiazepines highly did so because it is included in Beers Criteria [51], a consensus-developed list of potentially inappropriate medications in the elderly, and because, as one expert stated, it is “likely to have withdrawal” effects. Some experts also considered this group of drugs to be “most difficult to convince patients or physicians when they feel it is not causing a problem or if other alternatives not as effective”. Similarly, comments related to tricyclic antidepressants and other drug/drug classes rated as high priorities seemed to focus on the potential for adverse events and on being on the Beers Criteria list.

No participant rated bisphosphonates, zopiclone or trazodone as the highest priority (i.e. a score of one). Zopiclone and trazodone were the two drugs that were excluded at the end of round two.

### Round Three

Of the round three surveys sent to the 53 round two responders, 47 were completed. Therefore, 47 of the 65 (72%) round one participants (including the respondent who incorrectly completed round 1 but completed rounds 2 and 3 correctly) completed the survey in round three. We received one automatic reply indicating the recipient was away during the timeframe allotted to complete the round three survey. Table 5 shows the final rankings of all 14 drug classes included in round three in the order of number of respondents choosing that class for their top five priorities for deprescribing guidelines. In the event of a tie, the drug class with the lower mean rank is considered the higher priority. Benzodiazepines and atypical antipsychotics remained at the top of the priority list for most participants, followed by statins, tricyclic antidepressants and proton-pump inhibitors.

**Table 5:** Round Three Ranking: by number of participants who indicated drug class was a high priority for deprescribing guideline development.

Rank	Drug	Number of participants who indicated drug class was a high priority (%)	Mean	Standard deviation
**#1**	Benzodiazepines	43/47 (91%)	1.49	0.87
**#2**	Atypical antipsychotics	38/47 (81%)	2.32	1.05
**#3**	Statins	22/47 (47%)	3.14	1.22
**#4**	Tricyclic antidepressants	21/47 (45%)	3.29	1.16
**#5**	Proton-pump inhibitors	20/47 (43%)	3.5	0.92
**#6**	Urinary anticholinergics	17/47 (36%)	3.82	1.15
**#7**	Typical antipsychotics	16/47 (34%)	3.38	0.93
**#8**	Cholinesterase inhibitors	16/47 (34%)	3.88	1.32
**#9**	Opioids	12/47 (26%)	3.42	1.5
**#10**	Selective serotonin reuptake inhibitors	9/47 (19%)	4.11	1.1
**#11**	Bisphosphonates	8/47 (17%)	3.75	1.3
**#12**	Anticonvulsants	7/47 (15%)	4.14	0.83
**#13**	Beta-blockers	3/47 (6%)	4	1.41
**#14**	Antiplatelets	3/47 (6%)	5	0

## Discussion

Adults, especially elderly adults, often live with chronic diseases that are managed with multiple medications [5]. Health care providers work in a culture that facilitates diagnosing and prescribing, and that pays relatively little attention to deprescribing or reducing chronic medications. This can lead to overtreatment and drug-related illness [2]. Our Delphi consensus process mobilized experienced practitioners who care for the elderly, to identify drug classes in need of guidelines to assist with the deprescribing of medications that are no longer needed or may be causing problems. With these priorities in mind, we are able to move ahead in developing approaches to address overtreatment in the elderly and ultimately improve patient care.

A central goal of our guideline development project is to develop strategic evidence-based deprescribing guidelines to improve patient outcomes [43]. The drug/drug class selection focused on perceived need (considering a patient scenario), clinical gaps and usefulness for practitioners. We thus selected practitioners who work with older patients with chronic illness: primary care physicians, geriatricians, pharmacists, and nurse practitioners. Using practitioners to select drug/drug classes ensured that our process for selecting topics for guidelines was sensitive to the needs of the future guideline users, and that the drug/drug classes we selected represented important clinical deprescribing challenges. However, in working with the perceived needs of guideline users we risked a selection bias [52] (which could potentially lead to overemphasizing newer drugs such as atypical antipsychotics and underemphasizing older drug classes such as tricyclic antidepressants). Surveying and synthesizing rankings from practitioners across Canada could also result in a loss of precision in terms of potential local prescribing trends. Significantly more pharmacists than physicians and nurses participated in, and completed all survey waves. More than 50% of physicians and 40% of nurse practitioners dropped out, while more than 90% of pharmacists completed all surveys. The findings showed the participating pharmacists were most committed to the Delphi process and had a strong influence on the final ranking. It is unclear if the final priorities identified would have been different if more physicians or nurse practitioners had participated or completed all waves. The Delphi consensus process ultimately allowed the research team to narrow down drug/drug classes for guideline development that reflect the needs and priorities of practitioners working with older adults. The low agreement on the ranking of classes that was observed in round two may reflect the large number of drug classes that experts feel could benefit from the development of deprescribing guidelines and clinician bias towards those commonly seen in their own practices. Indeed, the qualitative comments provided insight into respondents thought processes in assigning particular ratings and demonstrate the significant variation in practitioners’ needs and wants for deprescribing guidelines. The classes of drugs that ultimately emerged as priorities from the final rankings dealt with mental health, cardiovascular, gastroenterological, and neurological conditions. Three of the five drug classes selected as highest priority dealt with mental health conditions. Benzodiazepines stood out in the consensus with the number one ranking in all three waves and atypical antipsychotics also retained a high rank across the three rounds. Both of these medication classes appear on the Beers Criteria, and Delphi participants commented on both the potential for adverse events and the withdrawal effects of deprescribing. Analysis of public drug program expenditures in Canada demonstrate that 21% of seniors had at least one claim for a benzodiazepine-type drug in 2009–2010 [53], despite recommendations to minimize their use due to risk of adverse effects [54] and the existence of effective approaches to reducing their use [55]. Given the prevalence of use, it’s not surprising that this group of medications consistently rank as the number one priority for deprescribing guidelines. While effective approaches to discontinuation exist, clinicians clearly still need assistance with negotiating changes with patients, finding non-pharmacologic approaches to manage symptoms and managing the process of tapering. While antipsychotic use is not as prevalent (5% of seniors in Canada having had a claim in 2009–2010) [53], concern over limited effectiveness for neuropsychiatric symptoms in dementia and potential adverse effects, including higher mortality with long-term use [56], is likely prompting clinicians desire for guidance in stopping these agents.

In our final wave, statins ranked 3^rd^ and based on respondent comments, this may be related to concerns about benefit given the lack of elderly patients in trials, lack of clarity around ongoing indication and when they can be stopped, as well as an emerging recognition of side effects and overtreatment of low risk patients [57,58]. Tricyclic antidepressants ranked 4^th^ and based on respondent comments, this may be related to both challenges in stopping them, for example, managing family physician reluctance to alter sleep or mood stability while trying to limit risk, for example, known side effects such as confusion and falls [59]. Finally, proton pump inhibitors, which ranked 5^th^, were seen by respondents as being overused and relatively easy to stop despite concerns over symptom recurrence [60–62].

In addition to the top five drug classes outlined above, the Delphi process identified nine other drug classes in need of evidence-based guidelines (see Table 4). These include treatments for a range of mental health and chronic disease conditions, often highlighting drug classes where prolonged use has recently come into question (e.g. bisphosphonates, anticonvulsants etc) [63,64], drug classes where a specialist may have initiated therapy but a primary care practitioner needs to determine ongoing need (e.g. beta-blockers, antiplatelets, cholinesterase inhibitors), or symptomatic treatments where ongoing benefit versus harm of the medication remains in doubt (e.g. urinary anticholinergics, opioids, serotonin reuptake inhibitors, typical antipsychotics). This broad range of drug classes suggests that overtreatment occurs through the full spectrum of primary care. While we were able to rank drug classes in order, there was little agreement among respondents regarding priorities for evidence-based deprescribing guidelines. There could be many reasons for this including differences in local prescribing needs, patient or health system barriers or perhaps personal self-efficacy for deprescribing tasks for certain drug classes. All of these are worthy of further study. Continuing to develop evidence-based deprescribing guidelines remains a priority; however, given the wide range of drug classes identified as needing evidence-based guidelines, we recognize that it would be prudent for all chronic disease and mental illness disease guidelines to include deprescribing components.

We note that all participants from our Delphi process were Canadian clinicians, and that the resulting priorities reflect the conditions, drug benefit plans, experiences and judgments of prescribers operating within the multi-jurisdictional Canadian health system. Canada has a single payer universal access health system but it does not have a universal drug coverage plan. It is likely that deprescribing priorities are influenced by prescribing patterns, health conditions, and health system pressures that arise within specific contexts in different geographies and countries.

## Conclusions

This Delphi consensus process helped to identify and prioritize the five medication classes that clinicians believed would most benefit from deprescribing guidelines. The classes of drugs that emerged strongly from the rankings dealt with mental health, cardiovascular, gastroenterological, and neurological conditions. The process also identified nine other drug classes in need of evidence-based guidelines, including treatments for a range of chronic disease conditions. The results suggest that deprescribing and overtreatment occur through the full spectrum of primary care, and that the development of evidence-based deprescribing guidelines, and the inclusion of deprescribing components in all chronic disease guidelines, are a priority in the care of the elderly.

## Supporting Information

S1 File Delphi survey round 1. (DOCX)

S2 File Delphi survey round 2. (DOCX)

S3 File Delphi survey round 3. (DOCX)
