# Supplementary material for: What Are Priorities for Deprescribing for Elderly Patients? Capturing the Voice of Practitioners: A Modified Delphi Process
Source: PLoS One. 2015 Apr 7;10(4):e0122246. doi: 10.1371/journal.pone.0122246 (PMC4388504; doi:10.1371/journal.pone.0122246)
Supplement: S1 File — (DOCX) [file pone.0122246.s001.docx]

**Appendix S1:** Delphi survey round 1

**Delphi Expert Consensus Survey: Identifying Priorities for Deprescribing Guidelines**

**Introduction and Instructions**

Welcome! Thank you for agreeing to participate in our Delphi Consensus Priority setting process.  We value your judgment and we appreciate your participation in this process.

We plan to develop up to five evidence based ‘deprescribing guidelines’ to help clinicians determine the appropriateness of stopping medications in the elderly (> 65 years of age), to facilitate tapering and stopping medications, as well as monitoring for and managing adverse events that might occur when stopping the medication.  Your expertise and experience will help us identify categories of medications for which ‘deprescribing guidelines’ would be useful.

Imagine you are a relatively new clinician working in a primary care or long-term care practice with a number of elderly people.   You have several patients for whom you question the clinical effectiveness of, and are concerned about side effects from medications.  Based on estimates of harm vs. benefit, you’d like to stop some medications but would appreciate guidance regarding which ones should be discontinued and how this should be done.

Thinking about the following four criteria commonly used in guideline development, (adapted from the GRADE guideline development approach), please rank each medication or medication class on a scale from 1 to 5, indicating the ’usefulness’ of an evidence based ‘deprescribing guideline’ to help stop a medication in the above scenario.

1. Weighing benefits vs. harms of medication therapy (e.g. where harm of continuing the medication, such as actual or potential adverse effects, or contribution to  pill burden, might outweigh benefit)
2. Certainty of estimate of effects (e.g. where  benefit for continued use of the medication is uncertain)
3. Patient preference and values (e.g. is it acceptable to patients?)
4. Feasibility (e.g. from both patient and physician perspective) and cost (e.g. cost savings to the system or patient)

Your assessment of ‘usefulness’ should also consider the need to have guidance in both stopping the medication and managing the impact of stopping the medication.   
 
There are 4 sections to the survey.  Section 1 includes demographics.  The medications and classes that comprise Section 2 of the survey were chosen after reviewing the literature on potentially inappropriate medications in the elderly or the frail elderly, the propensity for adverse events and related emergency department and hospital admissions, as well as medication utilization and costs in the elderly. We recommend you review the table below in its entirety before you begin ranking each medication/class.   There is an open-ended comment box at the end of each row if you have optional comments you’d like to share with us.  In Section 3 of the survey you will find space to add medications or classes that are not included in Section 2, and which you would like us to consider. The last section provides information regarding how you can become involved in the Deprescribing project.

After we receive feedback from a majority of participants, we’ll send you a summary of the group responses and include priority medications or classes identified in the first survey so that you may compare your own priorities with those identified by others, and revise your priorities if you so desire.  This will help us build consensus about what guidelines would be most useful to clinicians and most useful to building a foundation for the field of evidence based deprescribing.

**Section 1: Demographics**

*Role(s) (check all that apply):*

Pharmacist

Pharmacist (Certified Geriatric Pharmacist)

Academic Pharmacist (research, university teaching)

Family Physician

Family Physician (Care of the Elderly)

Geriatrician

Nurse Practitioner

Policy Decision Maker

Other ________________________

*Years of experience working with patients over 65:*

Less than 5 yrs  5 – 9  10 – 14  15 – 19  20 – 24  25 yrs or more

*Practice Type*

Long-Term Care  Primary Health Care  Other  Retired

*Which of the following best describes how you identify yourself:*

Male  Female  Other (e.g. transgender)

*Age:*

Under 3435– 4445 – 5455 - 6465 and older

*Province/Territory*

BC  AB  SK  MB  ON  QC  NB  NS  NL  PE

YT  NT  NU

**Section 2: Prioritizing Drugs/Drug Classes for Deprescribing Guideline Development**

We recommend you review the entire list of drug classes/drugs below before you begin ranking each class/drug.

Reminder:

Thinking about the four criteria described earlier, please rank each medication or medication class on a scale from 1 to 5, indicating the ’usefulness’ of an evidence based ‘deprescribing guideline’ to help stop a medication.

1. Weighing benefits vs. harms of medication therapy
2. Certainty of estimate of effects
3. Patient preference and values
4. Feasibility and cost

Acronyms used:
- NSAIDs: Non-Steroidal Anti-inflammatory Drugs

- SSRIs: Selective Serotonin Reuptake inhibitors
- TCAs: Tricyclic Antidepressants
- ACEIs: Angiotensin Converting Enzyme Inhibitors
- ARBs: Angiotensin Receptor Blockers
- CCBs: Calcium Channel Blockers

|  | Evidence-based deprescribing guidelines for this drug class or for a specific drug in this class will be: | | | | | Comments |
| --- | --- | --- | --- | --- | --- | --- |
| Drug class or Drug | Definitely useful | Probably useful | Might be useful | Likely not useful | Definitely not useful |  |
|  | 1 | 2 | 3 | 4 | 5 |  |
| Analgesics | | | | | |  |
| - NSAIDS |  |  |  |  |  |  |
| - Opioids |  |  |  |  |  |  |
| Anticoagulants |  |  |  |  |  |  |
| Antidepressants | | | | | |  |
| - Selective serotonin reuptake inhibitors (SSRIs) |  |  |  |  |  |  |
| - Tricyclic antidepressants (TCAs) |  |  |  |  |  |  |
| Antihypertensives |  |  |  |  |  |  |
| - Angiotensin converting enzyme inhibitors (ACEIs) |  |  |  |  |  |  |
| - Angiotensin receptor blockers (ARBs) |  |  |  |  |  |  |
| - Beta-blockers |  |  |  |  |  |  |
| - Calcium channel blockers (CCBs) |  |  |  |  |  |  |
| - Diuretics |  |  |  |  |  |  |
| Antiplatelet agents |  |  |  |  |  |  |
| Antipsychotics | | | | | | |
| - Typical antipsychotics |  |  |  |  |  |  |
| - Atypical antipsychotics |  |  |  |  |  |  |
| Benzodiazepines |  |  |  |  |  |  |
| Cholinesterase inhibitors |  |  |  |  |  |  |
| Digoxin |  |  |  |  |  |  |
| Glucocorticoids |  |  |  |  |  |  |
| Hypoglycemics | | | | | | |
| - Glyburide |  |  |  |  |  |  |
| - insulin |  |  |  |  |  |  |
| - metformin |  |  |  |  |  |  |
| Non-benzodiazepine drugs used as sedatives | | | | | | |
| - barbiturates |  |  |  |  |  |  |
| - zopiclone |  |  |  |  |  |  |
| - trazodone |  |  |  |  |  |  |
| - oral antihistamines |  |  |  |  |  |  |
| Proton pump inhibitors (PPIs) |  |  |  |  |  |  |
| Statins |  |  |  |  |  |  |
| Tamoxifen |  |  |  |  |  |  |
| Urinary anticholinergics |  |  |  |  |  |  |
| Vitamins |  |  |  |  |  |  |

**Section 3: Identifying Additional Priorities for Deprescribing Guideline Development**

Please add any medications or medication classes for which you feel deprescribing guidelines would be beneficial and explain why.

|  |
| --- |

Thank-you for participating in this Delphi Expert Consensus process. We will analyze the results and provide you with the group rating, along with your original rating within the next month.
