# Supplementary material for: What Are Priorities for Deprescribing for Elderly Patients? Capturing the Voice of Practitioners: A Modified Delphi Process
Source: PLoS One. 2015 Apr 7;10(4):e0122246. doi: 10.1371/journal.pone.0122246 (PMC4388504; doi:10.1371/journal.pone.0122246)
Supplement: S2 File — (DOCX) [file pone.0122246.s002.docx]

**Appendix S2:** Delphi survey round 2

**Delphi Expert Consensus Survey**

**Prioritizing Topics for Deprescribing Guideline Development for the Elderly – Round 2**

**Introduction**

Thank you for participating in Round 1 of the Delphi consensus priority setting process for deprescribing guidelines for the elderly.

There are two sections in the Round 2 survey. Section 1 includes new drug/drug classes that were added by ≥10% of the respondents in Round 1. You will be asked to rate these additional drugs using the same process that you used to rate drugs/drug classes in Round 1. Section 2 includes those drug/drugs classes that were identified by 70% of the Round 1 respondents as ranking either 4 – Probably useful, or 5 – Definitely useful, as well as the two new drug classes added by the Round 1 respondents. In this section, you will be asked to rank the list of drugs/drug classes from 1-16.

**Instructions for Section 1: Prioritizing Newly Identified Drugs/Drug Classes for Deprescribing Guideline Development**

Imagine you are a relatively new clinician working in a primary care or long-term care practice with a number of elderly people.   You have several patients for whom you question the clinical effectiveness of and are concerned about side effects from medications.  Based on estimates of harm vs. benefit, you’d like to stop some medications but would appreciate guidance regarding which ones should be discontinued and how this should be done.

Thinking about the following four criteria commonly used in guideline development, (adapted from the GRADE guideline development approach), please rank each medication or medication class on a scale from 1 to 5, indicating the ’usefulness’ of an evidence based ‘deprescribing guideline’ to help stop a medication in the above scenario.

1. Weighing benefits vs. harms of medication therapy (e.g. where harm of continuing the medication, such as actual or potential adverse effects, or contribution to pill burden, might outweigh benefit)

2. Certainty of estimate of effects (e.g. where benefit for continued use of the medication is uncertain)

3. Patient preference and values (e.g. is it acceptable to patients?)

4. Feasibility (e.g. from both patient and physician perspective) and cost (e.g. cost savings to the system or patient)

Your assessment of ‘usefulness’ should also consider the need to have guidance in both stopping the medication and managing the impact of stopping the medication.

|  | Deprescribing guidelines for this drug class or for a specific drug in this class will be: | | | | | Comments |
| --- | --- | --- | --- | --- | --- | --- |
| Drug or Drug class | Definitely not useful | Likely not useful | Might be useful | Probably useful | Definitely useful |  |
|  | 1 | 2 | 3 | 4 | 5 |  |
| Anticonvulsants |  |  |  |  |  |  |
| Bisphosphonates |  |  |  |  |  |  |

**Instructions for Section 2: Ranking Drugs/Drug Classes for Deprescribing Guideline Development**

Section 2 includes those drug/drugs classes that were identified by 70% of the Round 1 respondents as ranking either 4 – Probably useful, or 5 – Definitely useful, as well as the two new classes of drugs added by the Round 1 respondents. The latter will be removed from the analysis should they not meet the criteria of 70% of respondents indicating them to be probably or definitely useful.

Please rank the following drug/drug classes in order of priority from 1 to 16 with respect to the need for a deprescribing guideline (eg: Ranking #1 indicates highest priority for the need of a deprescribing guideline).
 
Please include, for the top 5 ranked drugs/drug classes, your reasons for ranking these as highest priority. This justification will help the research team better understand why a drug/drug class was considered a higher priority than others and will aid in breaking ties between drugs/drug classes if necessary.

**Note**: Drug/Drug Classes are presented alphabetically.

| Drug or Drug Class (presented alphabetically) | Your Ranking (1 = highest priority. 16 = lowest priority) | Justification (for the Top 5 drugs) |
| --- | --- | --- |
| Analgesics - Opioids |  |  |
| Anticonvulsants |  |  |
| Antidepressants (SSRIs) |  |  |
| Antidepressants (TCAs) |  |  |
| Antihypertensives (Beta Blockers) |  |  |
| Antiplatelet Agents |  |  |
| Antipsychotics (Typical) |  |  |
| Antipsychotics (Atypical) |  |  |
| Benzodiazepines |  |  |
| Bisphosphonates |  |  |
| Cholinesterase inhibitors |  |  |
| Non-benzodiazepine drugs used as sedatives: Trazodone |  |  |
| Non-benzodiazepine drugs used as sedatives: Zopiclone |  |  |
| Proton Pump Inhibitors |  |  |
| Statins |  |  |
| Urinary Anticholinergics |  |  |

**Thank-you for participating in Round 2 of the Delphi consensus priority setting process to identify priorities for deprescribing guidelines for the elderly. Following analysis of the results of Round 2 we will determine whether or not a third round of surveys is necessary. Regardless, the final results will be shared with all respondents.**
