# Supplementary material for: What Are Priorities for Deprescribing for Elderly Patients? Capturing the Voice of Practitioners: A Modified Delphi Process
Source: PLoS One. 2015 Apr 7;10(4):e0122246. doi: 10.1371/journal.pone.0122246 (PMC4388504; doi:10.1371/journal.pone.0122246)
Supplement: S3 File — (DOCX) [file pone.0122246.s003.docx]

**Appendix S3:** Delphi survey round 3

**Delphi Expert Consensus Survey: Prioritizing Topics for Deprescribing Guideline Development for the Elderly – Round 3**

**Introduction**

Thank you for participating in Round 2 of the Delphi consensus priority setting process for deprescribing guidelines for the elderly.

As you know, we are developing and launching a new type of evidence-based guideline for deprescribing for the elderly. In order to have the greatest impact on patient care, we are trying to address the **urgent and clear needs** of health care providers practicing across Canada.

As such, in this round we are asking you to identify the top five drug classes that you feel **urgently require a guideline.**

**Ranking the Top Five priority drug classes for Deprescribing Guideline Development**

The following table includes the 14 drug classes from Round 2. Two individual drugs (trazodone and zopiclone) have been removed because the research team would like to prioritize guidelines that can be used for drug classes, rather than individual drugs.

The drug classes are presented in priority order, as identified by Delphi participants in Round 2. The mean rank represents the average rank participants assigned each particular drug class. Therefore, the lowest mean rank signifies the highest priority drug class.

From feedback provided in Round 2, we identified five key reasons why respondents felt many of the drug classes listed required deprescribing guidelines.  Please consider these five criteria while prioritizing drug classes in this round:

- uncertainty of benefit in the elderly,
- high risk of harms in the elderly,
- availability of suitable alternatives,
- potentially high impact of a deprescribing guideline for the elderly,
- feasibility for guideline development (adequate amount of literature to create an evidence-based guideline).

You will now be asked to rank your top five priorities for urgent development of deprescribing guidelines. You may be influenced by the five criteria above, by how the expert group has rank ordered the drug classes in Round 2 (see table below) and by your own experience.

| Rank | Drug Class | Mean Rank | Standard Deviation |
| --- | --- | --- | --- |
| Highest priority | Benzodiazepines | 3.08 | 2.84 |
|  | Atypical Antipsychotics | 5.58 | 4.15 |
|  | Tricyclic Antidepressants | 7.38 | 3.55 |
|  | Typical Antipsychotics | 7.72 | 4.6 |
|  | Statins | 7.98 | 4.49 |
|  | Proton-Pump Inhibitors | 8.04 | 4.7 |
|  | Cholinesterase Inhibitors | 8.58 | 4.54 |
|  | Opioids | 8.62 | 5.09 |
|  | Urinary Anticholinergics | 8.91 | 4.48 |
|  | Selective serotonin reuptake inhibitors | 9.53 | 3.91 |
|  | Bisphosphonates | 9.83 | 3.69 |
|  | Beta Blockers | 10 | 4.07 |
|  | Anticonvulsants | 10.38 | 4.32 |
| Lowest priority | Antiplatelets | 10.87 | 3.85 |

**Final Ranking**

Taking into consideration the ranking of drug classes from the last survey, and considering these criteria:

- uncertainty of benefit in the elderly,
- high risk of harms in the elderly,
- availability of suitable alternatives,
- potentially high impact of a deprescribing guideline for the elderly,
- feasibility for guideline development (adequate amount of literature to create an evidence-based guideline),

my top five drug priorities for the urgent development of deprescribing guidelines for the elderly are:

#1: __________________

#2: __________________

#3:___________________

#4:___________________

#5: ___________________

Thank-you for participating in Round 3 of the Delphi consensus priority setting process for deprescribing guidelines for the elderly.
